# Long-term starvation and ageing induce AGE-1/PI 3-kinase-dependent translocation of DAF-16/FOXO to the cytoplasm

**DOI:** 10.1186/1741-7007-4-1

**Published:** 2006-02-03

**Authors:** David Weinkove, Jonathan R Halstead, David Gems, Nullin Divecha

**Affiliations:** 1 Department of Biology, University College London, Gower Street, London WC1E 6BT, UK; 2 Division of Cellular Biochemistry, Netherlands Cancer Institute, Plesmanlaan 121, 1066 CX, Amsterdam, The Netherlands

## Abstract

**Background:**

The provision of stress resistance diverts resources from development and reproduction and must therefore be tightly regulated. In *Caenorhabditis elegans*, the switch to increased stress resistance to promote survival through periods of starvation is regulated by the DAF-16/FOXO transcription factor. Reduction-of-function mutations in AGE-1, the *C*. *elegans *Class IA phosphoinositide 3-kinase (PI3K), increase lifespan and stress resistance in a *daf-16 *dependent manner. Class IA PI3Ks downregulate FOXOs by inducing their translocation to the cytoplasm. However, the circumstances under which AGE-1 is normally activated are unclear. To address this question we used *C. elegans *first stage larvae (L1s), which when starved enter a developmentally-arrested diapause stage until food is encountered.

**Results:**

We find that in L1s both starvation and *daf-16 *are necessary to confer resistance to oxidative stress in the form of hydrogen peroxide. Accordingly, DAF-16 is localised to cell nuclei after short-term starvation. However, after long-term starvation, DAF-16 unexpectedly translocates to the cytoplasm. This translocation requires functional *age-1*. H_2_O_2 _treatment can replicate the translocation and induce generation of the AGE-1 product PIP_3_. Because feeding reduces to zero in ageing adult *C. elegans*, these animals may also undergo long-term starvation. Consistent with our observation in L1s, DAF-16 also translocates to the cytoplasm in old adult worms in an *age-1*-dependent manner.

**Conclusion:**

DAF-16 is activated in the starved L1 diapause. The translocation of DAF-16 to the cytoplasm after long-term starvation may be a feedback mechanism that prevents excessive expenditure on stress resistance. H_2_O_2 _is a candidate second messenger in this feedback mechanism. The lack of this response in *age-1(hx546) *mutants suggests a novel mechanism by which this mutation increases longevity.

## Background

A widely held view in ageing research is that the rate of ageing is influenced by the division of resources between growth rate and reproduction on one hand, and somatic maintenance on the other [1]. This theory proposes that evolution sets the level of somatic maintenance to be just sufficient to prevent damage that would interfere with growth and reproduction but not waste energy that could be otherwise used to produce offspring or increase the rate of development. Thus, rather than being an active programme, ageing results from the eventual failure of the somatic maintenance set by this balance. However, the balance of resources between somatic maintenance and reproduction must also be responsive to the environment because external conditions affect growth and development and can vary considerably. Understanding this plasticity may help us understand how ageing is regulated and is subject to external interventions.

In the nematode *Caenorhabditis elegans*, the FOXO transcription factor DAF-16 plays a major role in mediating changes in resource allocation in response to environmental change. Firstly, DAF-16 is required for entry into the diapausal (developmental and growth arrested) dauer stage under conditions of starvation and overcrowding [2]. Formation of this stress-resistant alternative to the normal third larval stage (L3) involves several morphogenetic and metabolic changes and upregulation of somatic maintenance genes. Dauer larvae can survive for several months until they find food, upon which they resume normal development into adults that have a normal lifespan [3].

Secondly, outside its role in dauer formation, DAF-16 activity is associated with increased stress resistance and survival and reduced reproductive fitness [4]. Most studies of DAF-16 have focussed on its role in mediating the phenotypes of mutants of the *C. elegans *insulin/IGF like signalling (IIS) pathway. Activation of this pathway leads to the translocation of DAF-16 from the nucleus to the cytoplasm. When this pathway is disrupted by mutations such as reduction-of-function mutants of the *daf-2 *insulin-like receptor or the *age-1 *Class IA phosphoinositide 3-kinase (PI3K), the propensity to form dauers, stress resistance and adult lifespan are all increased as a result of DAF-16 activation [3,5,6]. Studying gene expression in these mutants has led to the identification of many genes upregulated by DAF-16 [7-9]. These genes are involved in several processes that may increase lifespan, including those clearly involved in somatic maintenance such as heat shock proteins and antioxidant proteins. Consistent with the cost of DAF-16 activation, long-lived *daf-2 *animals show reduced fertility and cannot compete with the wild type [10,11]. Furthermore, overexpression of DAF-16 using transgenes slows development [12,13] and *daf-16 *reduction of function by RNAi or mutation causes worms to reach reproductive adulthood slightly before wild type animals ([4], unpublished observations), suggesting that even in the wild type, DAF-16 acts to slow development.

In contrast to *daf-2 *mutants, the long-lived *age-1(hx546) *mutant is able to compete with the wild type over several generations when the two strains are mixed under standard culture conditions [14]. However, the *age-1(hx546) *mutant loses the ability to compete with the wild type if the mixed population is exposed to repeated cycles of starvation and feeding [14]. These competition experiments are very sensitive to small changes in fitness and suggest that the *age-1(hx546) *mutation does not cause constitutive activation of DAF-16 but interferes with the ability of the worm to adapt to changing conditions. In agreement with this conclusion, a study of DAF-16 localisation reported no effect of the *age-1(hx546) *mutation under normal culture conditions [4]. *age-1(hx546) *is a reduction-of-function mutant rather than a null mutation and its molecular nature is unknown [15,16]. Interestingly, only with age do *age-1(hx546) *mutants become resistant to hydrogen peroxide and show elevated levels of superoxide dismutase and catalase activities [17,18]. In summary, the *age-1(hx546) *mutation appears to affect the animal only during starvation and in ageing adults, so understanding how AGE-1 regulates DAF-16 during these conditions may reveal how this mutation causes increased longevity.

In this study, we focus on the starvation-induced first larval stage (L1) diapause [Additional File 1]. Like the dauer stage, the L1 diapause allows *C. elegans *to survive periods of long-term starvation. Starved L1s survive several weeks and time in this condition has no effect on subsequent adult lifespan [19]. However, unlike the dauer diapause, there are no obvious morphological changes in the starved L1 that might protect the larvae from external stresses. We investigated how L1 larvae respond to oxidative stress and have shown that starvation induces resistance to H_2_O_2 _in a *daf-16*-dependent manner. Consistent with these results, DAF-16 was localised in cell nuclei after short-term starvation. However, after long-term starvation, DAF-16a::GFP translocated to the cytoplasm and this translocation was absent in the *age-1(hx546) *mutant. We show that a similar effect occurs in old worms as they become starved with age. These data suggest a novel mechanism underlying longevity in weak *age-1 *mutants and reveals an unexpected negative regulation of FOXO transcription by Class IA PI3Ks in response to starvation.

## Results and discussion

To investigate the function and regulation of DAF-16 under conditions in which *C. elegans *encounter starvation, we turned to the L1 diapause. This diapause state is entered once larvae hatch from their eggshells in the absence of food. These larvae arrest in development and are viable without food for weeks, re-entering normal development when food is encountered. Unlike the dauer diapause, the L1 diapause can be entered (and exited) by mutants lacking functional *daf-16*. This property makes the L1 diapause a physiologically relevant model for studying DAF-16 function and regulation in response to starvation and other stresses.

**Figure 1:**
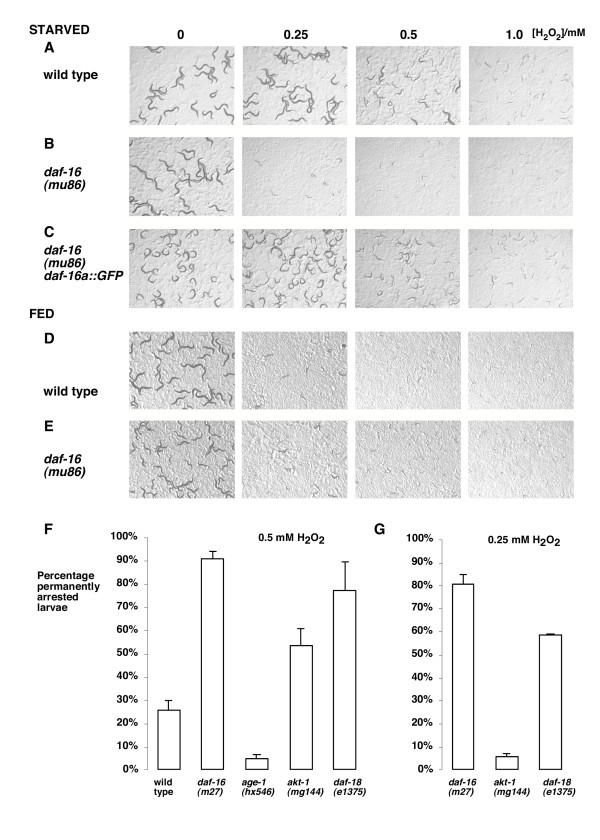
**Resistance to H_2_O_2 _requires *daf-16 *and starvation**. L1s with the indicated genotype were exposed to 0. 0.25 mM, 0.5 mM and 1 mM H_2_O_2 _for 45 minutes, washed and placed on agar plates containing bacterial food. Images were taken 48 hours later. Further examination revealed that the smaller larvae are alive but permanently arrested at around L2/L3 stage. (A-C) for larvae treated after hatching in liquid medium without food, mutation of *daf-16 *(B) causes larvae to be much more sensitive than the wild type (A) or *daf-16 *mutants rescued with the *muIs61 *transgene (C). (D, E) Larvae treated after hatching on NGM plates with bacterial food are hypersensitive to H_2_O_2_. (F.G) Mutations in the AGE-1/AKT pathway affect starvation-induced resistance to H_2_O_2_. Quantification of the proportion of permanently arrested larvae with treatments of 0.5 mM H_2_O_2 _(F) and 0.25 mM H_2_O_2 _(G) for the more sensitive strains (0.25 mM H_2_O_2 _had no effect on wild type larvae).

Most studies of *daf-16 *function have been carried out in the context of its role in the stress-resistance and extend lifespan of IIS mutants. Under standard growth conditions, mutation of *daf-16 *alone only slightly increases susceptibility to stress [20,21]. However, in this study we find that in the L1 diapause, mutation of *daf-16 *has a dramatic effect on the response of larvae to an acute treatment of hydrogen peroxide. For these experiments, we isolated L1s hatched in the absence of food in an aqueous phosphate buffer and exposed them to a range of concentrations of H_2_O_2 _for 45 minutes (see Methods). We then washed away the H_2_O_2 _and transferred the worms to agar plates containing bacterial food to reinitiate development and assess the long-term effects. The short-term effect of the treatment was to inhibit movement, but the major long-term effect was to cause an abnormal developmental arrest. For example, wild type larvae assessed two days after a treatment of 1 mM H_2_O_2 _remained permanently at a size corresponding to L2 or L3 stage and displayed very sluggish, uncoordinated movement. Starved L1s with loss-of-function mutations in *daf-16 *were very sensitive to H_2_O_2 _treatment: 0.25 mM H_2_O_2 _caused over 80% permanent arrest of *daf-16(m27) *or *daf-16(mu86) *mutants compared to no permanent arrest for the wild type (Figure 1B, F). The H_2_O_2 _sensitivity of *daf-16 *mutants was fully rescued by a transgene expressing a DAF-16a::GFP fusion protein [13] (Figure 1C), confirming the role of *daf-16 *in conferring H_2_O_2 _resistance.

To investigate the role of starvation, we tested the H_2_O_2 _resistance of L1s hatched in the presence of food on agar plates. In contrast to starved larvae, these fed larvae showed high sensitivity to 0.25 mM H_2_O_2 _(Figure ID) and it made little difference whether fed L1s were mutant for *daf-16 *or not (Figure 1E). These results demonstrate that *daf-16*-dependent protection of larvae from H_2_O_2 _depends on environmental conditions and is consistent with the hypothesis that DAF-16 is only activated when its transcriptional targets are needed for survival.

Mutation of the AGE-1 PI3K/Akt pathway affects *daf-16 *activity in dauer formation and lifespan. To test whether mutation of this pathway regulates the ability of *daf-16 *to protect starved L1s against H_2_O_2_, we quantified the resistance of mutants in the pathway using the assay described above. In order to assess how conditions and genotype affect H_2_O_2 _resistance, we used H_2_O_2 _concentrations that resulted in developmental arrest of a quantifiable proportion of the test population. For example, after treatment with 0.5 mM H_2_O_2 _approximately 30% of wild type larvae were permanently arrested at the L2/L3 stage, 20% were unaffected (Figure 1A, F and data not shown) and approximately 50% were temporarily delayed in development because they reached adulthood approximately one day later than did untreated controls.

Under these conditions, L1s with *age-1(hx546) *mutation showed increased resistance to H_2_O_2 _(Figure 1F). We next tested a gain-of-function mutation in the downstream kinase *akt-1 *[22] and a reduction-of-function mutation in *daf-18 *[23], the PIP_3 _3-phosphatase PTEN. These mutations activate the AGE-1 pathway and, in comparison to the wild type, they caused starved L1s to be more sensitive to H_2_O_2 _(Figure 1F). We also used a lower concentration of H_2_O_2 _(0.25 mM – which had no effect on the wild type) to differentiate these hypersensitive mutants further (Figure 1G). Thus, mutation of the AGE-1 pathway affects the ability of DAF-16 to protect worms from H_2_O_2_.

**Figure 2:**
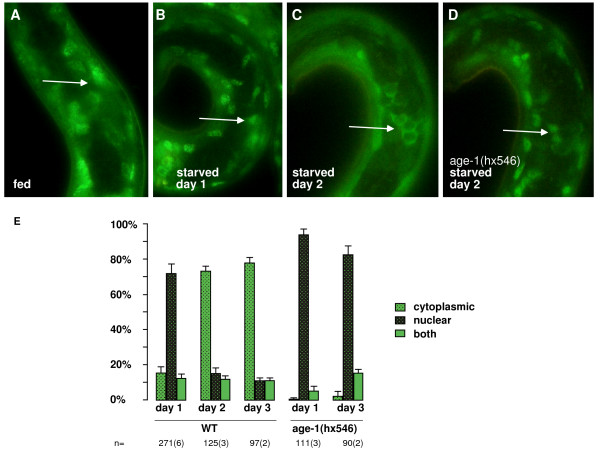
**Effects of starvation on DAF-16 localisation and activity. **(A-D) Images of live L1 larvae containing the *muIs71 *transgene expressing DAF-16a::GFP in a *daf-16(mu86) *background. Subcellular localisation of DAF-16a::GFP is clearest in the neurons of the nerve ring indicated by arrows. (A) fed larva, cytoplasmic and nuclear, (B) starved day 1, nuclear (C) starved day 2, cytoplasmic (with a characteristic "honeycomb" appearance) (D) *age-1(hx546) *starved day 2, nuclear (E) Quantification of the effect of prolonged starvation on DAF-16a::GFP localisation in wild type and *age-1(hx546) *L1 larvae. n= number of worms imaged. Number of independent experiments in brackets.

Although useful for identifying genes involved in a process, genetic analysis such as described above does not provide information on where, when or how genes are acting. To try to answer some of these questions, we investigated the subcellular localisation of DAF-16 because control of the presence of FOXO in the nucleus, where it acts as a transcription factor, is thought to be a major mechanism of its regulation. DAF-16 has been shown to translocate to the cell nucleus in response to heat stress, certain oxidative stresses and starvation [4,24]. To investigate how starvation and oxidative stress influence DAF-16 activity in our system, we examined DAF-16 localisation in L1 larvae, using a strain previously characterised in a study by Lin et al. in 2001 [13]. This strain contained the transgene *muIs71*, an integrated DAF-16a::GFP construct that rescues *daf-16 *activity. In L1s hatched in the presence of food, we found that, as previously reported, DAF-16a::GFP was located in both the nucleus and the cytoplasm [13] (Figure 2A). However, in over 70% of L1s hatched in liquid culture and starved for one day, DAF-16a::GFP was localised solely to the nucleus (Figure 2B, E). This result is consistent with the idea that localisation of DAF-16 in the nucleus is the mechanism by which starvation confers oxidative stress resistance.

**Figure 3:**
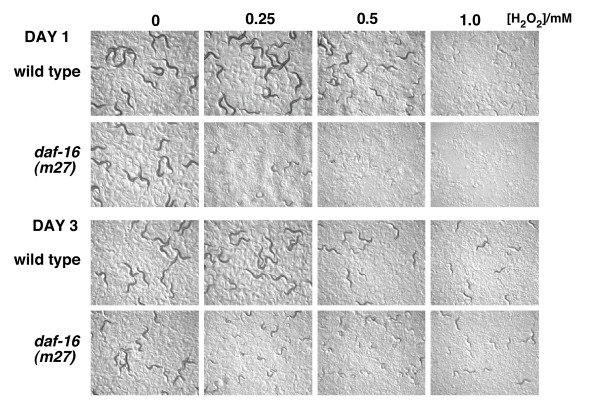
**Effect of prolonged starvation on resistance to H_2_O_2_**. Larvae were treated as in Figure 1, one or three days after egg isolation. Images of larvae were taken 48 hours after treatment.

However, in L1s starved for two days or more, we found that DAF-16a::GFP translocates back to the cytoplasm and was distinctly excluded from the nucleus in over 70% of larvae (Figure 2C and 2E). This translocation of the DAF-16 transcription factor to the cytoplasm upon long-term starvation might be expected to lead to a decrease in the transcription of target genes and therefore an increased sensitivity to H_2_O_2_-induced arrest. However, when we tested this hypothesis using the H_2_O_2 _resistance assay described above, we found that after three days of starvation larvae displayed levels of resistance similar to if not greater than those of larvae starved for one day (Figure 3). This resistance was still dependent on *daf-16 *activity because *daf-16 *mutants were still hypersensitive to H_2_O_2 _after three days (Figure 3). These results suggest that even though DAF-16 translocates to the cytoplasm upon prolonged starvation, sufficient *daf-16 *dependent transcription and translation of somatic maintenance genes has already occurred to provide long-term stress resistance. Thus, one possibility is that translocation to the cytoplasm after long term starvation prevents excess expenditure of resources on somatic maintenance processes.

A likely mechanism for the translocation of DAF-16 from the nucleus to the cytoplasm is the activation of the AGE-1 pathway. To test this possibility, we examined DAF-16a::GFP in starved L1s with the *age-1(hx546) *mutation. In these larvae DAF-16a::GFP remained in the nucleus, both on day one and during subsequent days of starvation (Figure 2D, E). Continuous localisation of DAF-16 to the nucleus in the *age-1(hx546) *mutants may increase allocation of resources to stress resistance. This increased expenditure might explain why this mutant is unable to compete with the wild type during cycles of feeding and starvation [14]. The *age-1 *dependent DAF-16 translocation we have discovered also suggests that long-term starvation activates AGE-1, which is a surprising conclusion considering current models proposing that Class IA PI3Ks are downstream targets of insulin-induced signalling in response to feeding. Recently a starvation-dependent, PI3K-dependent gene target has been identified in a human cancer cell line [25]. One possibility is that long-term starvation relieves a nutrition-induced feedback mechanism that downregulates PI3K signalling in mammalian cells [26].

**Figure 4:**
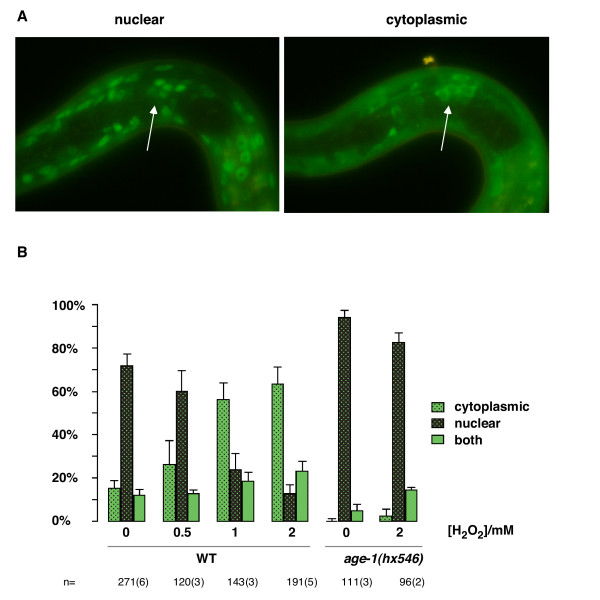
**H_2_O_2 _causes DAF-16a::GFP to translocate to the cytoplasm**. (A) Examples of *muIs71 daf-16(mu86) *worms, focussing on neurons of the nerve ring (arrows). DAF-16 is nuclear (after control treatment) or cytoplasmic (after H_2_O_2 _treatment). (B) Quantification of the effect of H_2_O_2_on DAF-16a::GFP localisation in *muIs71 daf-16(mu86) *worms with wild type *age-1 *and *age-1(hx546)*. n = number of worms imaged. Number of independent experiments in brackets.

*age-1*-dependent DAF-16 translocation in response to prolonged starvation might be a consequence of endocrine stimulation or might occur through the upregulation of a cellular signal. One candidate for such a signal is H_2_O_2 _itself, which has been shown to act as an intracellular second messenger. This molecule can activate the PI3K/AKT pathway to cause FOXO3a to translocate to the cytoplasm of mammalian cells [27]. To explore whether this response was conserved in *C. elegans*, we treated larvae starved for one day with increasing concentrations of H_2_O_2_. We found that H_2_O_2_, like long-term starvation, caused DAF-16a::GFP to translocate from the nucleus to the cytoplasm (Figure 4A, B). In a previous study, oxidative stress in the form of the superoxide producer juglone had been shown to cause DAF-16 to translocate to nucleus in fed L2 larvae [4]. We found that, in L1s starved for one day, neither juglone nor paraquat, another superoxide producer, had any significant effect on DAF-16a::GFP localisation (data not shown), possibly because DAF-16 is already mainly nuclear in these larvae. Thus, H_2_O_2 _has a different effect from superoxide producers on DAF-16 localisation.

**Figure 5:**
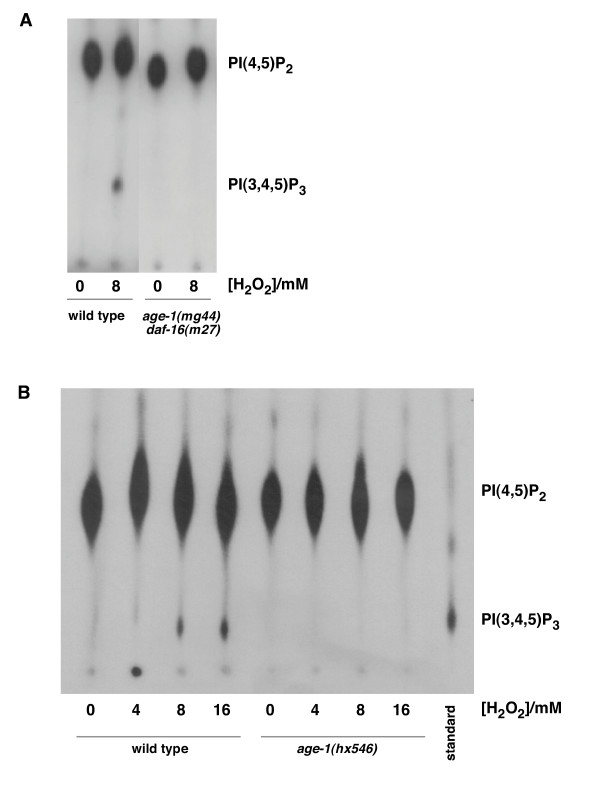
**H_2_O_2 _induces PIP_3 _production in an *age-1*-dependent manner**. Starved L1 larvae were ^32^P-orthophosphate labelled and the phosphoinositides were purified, deacylated and resolved using thin layer chromatography with a PIP_3 _standard. Autoradiographs show that (A) H_2_O_2 _induces PIP_3 _in wild type (N2) but not *age-1(mg44); daf-16(m27) *larvae. (B) Increasing concentrations of H_2_O_2 _causes increasing PIP_3 _in wild type (N2) larvae but no PIP_3 _is detectable in *age-1(hx546) *larvae.

Importantly, H_2_O_2_-induced translocation of DAF-16a::GFP was ablated in the *age-1(hx-546) *mutant (Figure 4B), suggesting that AGE-1 mediates DAF-16 translocation to the cytoplasm upon H_2_O_2 _treatment. To test the involvement of AGE-1 further, we developed techniques to determine whether phosphatidylinositol (3,4,5) trisphosphate (PIP_3_), the lipid product of AGE-1, is actually produced in *C. elegans *in response to H_2_O_2_. We found that, as in mammals, H_2_O_2 _did indeed stimulate PIP_3 _production in starved *C. elegans *L1s (Figure 5A). No PIP_3 _was produced in *age-1(mg44) *null mutants that were made viable by the *daf-16(m27) *loss-of-function mutation, demonstrating that PIP_3 _generation requires the AGE-1 Class IA PI3K. Next, we tested the long-lived *age-1(hx546) *mutant for PIP_3 _production. Despite the uncertainty about the molecular nature of this mutant, we found that no detectable PIP_3 _was produced in *age-1(hx546) *larvae after H_2_O_2 _stimulation (Figure 5B). Thus, in starved L1s, the *hx546 *mutation blocks the ability of the AGE-1 Class IA PI3K to produce PIP_3 _in response to H_2_O_2_.

Although it is technically difficult to test whether H_2_O_2 _is the endogenous signal that causes DAF-16 translocation to the cytoplasm after prolonged starvation, there have been several studies that make it a good candidate. Reactive oxygen species have been shown to increase upon serum or glucose starvation [27,28]. H_2_O_2 _can stimulate receptor tyrosine kinase signalling through its inhibitory effect on receptor tyrosine phosphatases. In addition, H_2_O_2 _can inhibit PTEN activity [29,30], stimulate the association of mammalian Class IA PI3Ks with Ras [31] and activate Type I PIP kinase dependent PtdIns(3,4)P_2_-5-kinase activity [32]. All the above mechanisms could lead to H_2_O_2_-induced PIP_3 _synthesis. Alternatively there could be a direct upregulation of IIS signalling in response to DAF-16 activation. A number of potential insulin-like ligands are upregulated in *daf-2 *mutants [7,9] and a recent study demonstrated that the *Drosophila *insulin receptor homologue was dramatically upregulated in flies overexpressing activated dFOXO [33].

**Figure 6:**
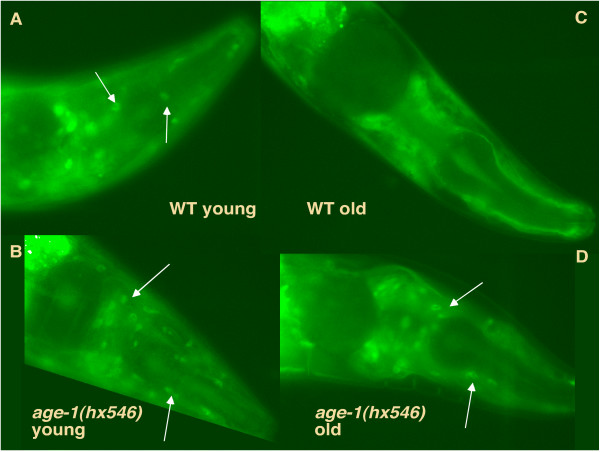
**DAF-16 translocates to the cytoplasm with age but not in *age-1(hx546) *mutants. **Images of DAF-16a::GFP localisation taken from live worms using a fluorescent compound microscope. Arrows indicate examples of distinct cell nuclei. (A) *age-1+ *3rd day of adulthood at 20°C (B) *age-1(hx546*) 2nd day of adulthood at 20°C (C) *age-1+ *8th day of adulthood (D) *age-1(hx546) *11th day of adulthood at 20°C. See Table 1 for more details.

As worms age, their pharyngeal pumping rate slowly reduces to zero [34]. We wondered whether this reduction of food intake causes long-term starvation and therefore investigated whether DAF-16 translocates to the cytoplasm of cells in old adult worms. The data published by Henderson and Johnson using a DAF-16::GFP fusion transgene suggest that in adults under standard laboratory conditions without starvation, the nuclear localisation of DAF-16 is not sufficient to discern nuclei. However, in the strain used in their study, DAF-16::GFP is expressed at noticeably higher levels than the strains used in this study, and they recently been reported that their transgene contains a missense mutation in the DAF-16 sequence [35]. Consistent with the initial description of the transgenes used in our study [13], we found that in reproductively active three day old adults raised at 20°C, DAF-16 was localised to both the cytoplasm and nuclei of cells with nuclei visible as distinct spots (Figure 6A).

However, as worms aged we found that nuclei became progressively less distinct, indicating a translocation to the cytoplasm (Figure 6C). In three day old worms carrying the *age-1(hx546) *mutation, DAF-16 localisation was similar to that seen in wild type worms of the same age, although nuclei were slightly more distinct than seen in the wild type (Figure 6B). However, in older *age-1(hx546) *mutants, DAF-16 became more nuclear and there was no translocation to the cytoplasm. Even in very old worms that were close to death as assessed by age, movement and appearance, cell nuclei were visible as distinct spots of GFP (Figure 6D, Table 1). Thus, there is an *age-1*-dependent translocation of DAF-16 to the cytoplasm in old worms. The lack of this age-dependent translocation in *age-1(hx546) *mutants may result in increased DAF-16 activity and hence explain the increased resistance to H_2_O_2 _and antioxidant activities observed in these mutants as they age [17,18].

Mice lacking the adaptor protein p66Shc, which binds to receptor tyrosine kinases, have an extended lifespan [36]. Interestingly, in cells derived from these mice, there is a defect in H_2_O_2_-induced translocation of FOXO3a to the cytoplasm [27]. We have demonstrated a physiological whole-organism situation in which a FOXO transcription factor translocates to the cytoplasm and shown that this response is defective in worms carrying the *age-1(hx546) *mutation, which has a strong effect on DAF-16 localisation only after prolonged starvation or ageing. Like this mutant, the p66Shc mutant mouse has increased stress resistance without obvious deleterious effects [36]. We propose a model whereby in these mutants, an evolutionarily-conserved response to prolonged starvation is traded for increased longevity.

Conclusion

**Figure 7:**
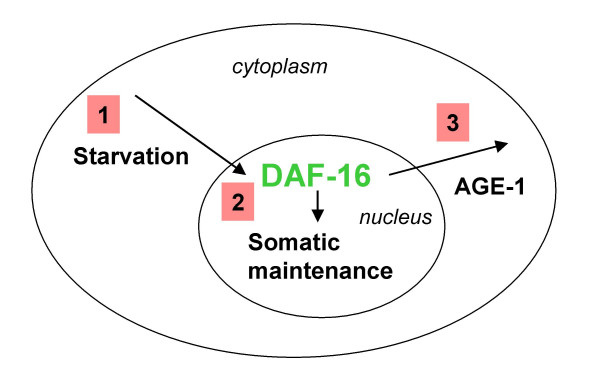
**Model of how starvation and AGE-1 regulate somatic-maintenance gene expression by regulating DAF-16 localisation. **Firstly, DAF-16 translocates to the nucleus in response to starvation. In the nucleus DAF-16 activates the transcription of somatic-maintenance genes until an unknown feedback mechanism causes the activation of AGE-1, which results in DAF-16 translocating back to the cytoplasm. This feedback is absent in *age-1(hx546) *mutants causing increased stress resistance in starved animals or ageing adults.

DAF-16/FOXO is nuclear-localised and active in the starved L1 diapause to enhance stress resistance but translocates back to the cytoplasm after long-term starvation in a manner dependent on *age-1/PI3K *(Figure 7). We propose this response is a negative feedback mechanism that prevents overexpenditure on resources during starvation. A similar effect is observed in adult worms. Disruption of this translocation may explain how the increased longevity of the *age-1(hx546) *mutant has no detrimental effects apart from the cost of competiveness during cycles of starvation and feeding [14].

## Methods

### Nematode culture and strains

Worms were cultured at 20°C on standard nematode growth medium (NGM), either using the *E. Coli *strain OP50 as food, or supplemented with 50μg/ml streptomycin and using the streptomycin-resistant OP50-1 strain as food. Strains used: N2, DR27 *daf-16(m27)*, TJ1052 *age-1(hx546)*, GR1310 *akt-1(mg144)*, CB1375 *daf-18(e1375*), XA2913 *age-1(mg44); daf-16(m27)*, CF1407 *muIs71 (daf-16a::gfp/bKO) + rol-6(su1006)]; daf-16(mu86)*, XA2950, *muIs61 [(daf-16a::gfp) + rol-6(su1006)]; daf-16(mu86) *outcrossed from CF1139 [13]; XA2954 *daf-16(mu86)*. The *age-1(mg44)*, *daf-16(m27) *and *daf-16(mu86) *alleles were tracked using single worm PCR and, where necessary, restriction digests. XA2974 and XA2975 (two independent strains) *age-1(hx546) muIs71 (daf-16a::gfp/bKO) + rol-6(su1006)]; daf-16(mu86) *were constructed using the strain XA2904. This strain contains a small stretch of chromosome II (around 2 map units from 1.23 to 3.27) that is derived from the Hawaiian strain CB4586. Because of extensive outcrossing, XA2904 contains no other CB4856 DNA as far as can be ascertained by SNP analysis. This strain was crossed into CF1407 and made homozygous for the polymorphic markers. It was then crossed to *age-1(hx546) *and made homozygous for N2 SNPs including the SNP pkP2110 [37], which is located within 0.35 map units of *age-1*.

### Assay for developmental stress effects

Starved L1s were isolated using the following method. Gravid adult worms in an excess of bacterial food were washed from 9 cm NGM agar plates into an Eppendorf tube using M9 buffer (41 mM Na_2_HPO_4_, 22 mM KH_2_PO_4_, 86 mM NaCl, 1 mM MgSO_4_). Adults were allowed to settle for a few minutes and the supernatant was removed until there was a volume of approximately 200 μl in the tube. 150 μl of bleach solution (7:8 sodium hypochlorite: 4 M NaOH) was added. The tube was shaken and monitored continually until the adults had dissolved (usually after 4 to 5 minutes). To stop the bleaching, the tube was filled up with M9 and centrifuged for one minute at 660 g. The supernatant was removed completely and replaced with fresh M9. The pellet was disassociated by rigorous shaking and the wash step repeated. The resulting eggs were left to hatch in liquid culture: 350–500 μl M9 buffer in a 24-well tissue culture plate. To make fed L1s, the eggs were spotted on NGM agar plates with a spot of OP50-1 *E. coli *food and used as soon as the majority of eggs had hatched (usually after 12–14 hours). Starved L1s in were used approximately 20 hours after bleaching. For the acute exposure to H_2_O_2_, 100–200 larvae were exposed to various concentrations of H_2_O_2_, dissolved in total volume of 500μl M9 in a 24-well plate. After approx 30 minutes, larvae were transferred to Eppendorf tubes and centrifuged at 2500 rpm for one minute. The supernatant was removed and replaced with M9, precisely 45 minutes after initial exposure to H_2_O_2_. The worms were sedimented again by centrifugation, the supernatant removed, and the worms placed on a spot of OP50-1 on NGM agar and placed in a 20°C incubator. Two days later the larvae were assessed for developmental stage. At this time point, untreated worms reached the L4 stage so effects on development could be assessed without the complication of adults producing progeny. L3 and L4 worms were scored as not permanently arrested (L3s at this time point are usually temporally arrested). Smaller worms were scored as permanently arrested.

### Visualising DAF-16a::GFP

To visualise DAF-16a::GFP in live L1 larvae, they were placed, after treatment, on a chilled agar pad on a microscope slide, covered with a glass coverslip and left for 10 to 30 minutes at 4°C to reduce movement. The slides were then examined under inverted fluorescence imaging. For each experiment we captured several images of the neurons in the head region where the subcellular localisation of DAF-16a::GFP was clear. These images were used to quantify differences in localisation between test populations. Adult worms were kept in cohorts to determine age and examined using inverted fluorescence imaging without anaesthetics.

### Detecting PIP_3 _in *C. elegans *larvae

Starved L1 larvae (prepared as described above starting with several large plates of adults) were washed into the phosphate-free "worm HEPES buffer (WHB)" (50 mM HEPES pH7.2, 100 mM NaCl, 1 mM MgSO_4_) and pooled so that there were 1–2 × 10^4 ^larvae in a 500 μl volume in a single well of a 24-well tissue culture dish. ^32^P orthophosphate (0.5 mCi, Amersham Biosciences) was added and the larvae were incubated at room temperature with gentle shaking for 4 hours. Larvae were transferred to tubes and washed twice with 15 ml WHB, resuspended in 250 μl WHB and put into a fresh 24 well plate in a total volume of 500 μl WHB with the appropriate concentration of H_2_O_2_. After the treatment, larvae were centrifuged in Eppendorf tubes and the supernatant removed to leave a total volume of approx 50 μl. Forty-five minutes after addition of H_2_O_2_, larvae were stopped with150 μl 2.4N HCl, 500 μl methanol and 250 μl chloroform containing lipid carrier (Folch Type I extract (Sigma) and 0.05% neomycin-purified lipids from Folch fraction). This single phase extraction was performed at room temperature with intermittent vortexing for 30 minutes. The contents of each tube were added to a 2 ml tube containing 250 μl chloroform/carrier, 100 μl 2.4N HCl and 150 μl H_2_O, and centrifuged. The bottom phase was removed and washed with theoretical upper phase. The lipids were extracted a second time with 500 chloroform/carrier and dried down. Lipids were deacetylated with methylamine and resolved on PEI cellulose plate using 0.45 M HCl as a solvent as described [32]. The PtdIns(3,4,5)P_3 _standard was generated by phosphorylating PtdIns(4,5)P_2 _with recombinant human p110alpha in the presence of ^32^Pγ ATP. Higher concentrations of H_2_O_2 _were needed to generate PIP_3 _reproducibly than were used to induce a developmental arrest in previous experiments because approximately 50 times more larvae were used for the biochemical analysis. These larger numbers of larvae required higher concentrations of H_2_O_2 _to induce developmental arrest (data not shown), probably because the larvae buffer H_2_O_2_. We have observed PIP_3 _production with as low as 1 mM H_2_O_2 _(data not shown) and increased PIP_3 _production with increasing H_2_O_2 _(Figure 5B).

## Abbreviations

PI3K – phosphoinositide 3-kinase, PIP_3 _– phosphatidylinositol (3,4,5) trisphosphate, WHB – worm HEPES buffer, PtdIns -phosphatidylinositol

## Authors' contributions

DW conceived of, designed and performed all the experiments and drafted the manuscript. JRH helped develop methods to isolate and measure PIP_3 _in *C. elegans *larvae. DG helped draft the manuscript. ND helped in the conception and design of the study. All authors have read and approved the manuscript.

## Supplementary Material

Additional File 1 **Diagram depicting the life cycle of *C. elegans *in the presence of food and in response to starvation**. The first stage larva hatches from the eggshell. In the presence of food the larva continues through four larval stages until it becomes a fertile adult. If the larva hatches in the absence of food it will enter the L1 diapause without undergoing the cell division cycles that normally occur in the L1 stage. When food is encountered, the larva will re-enter normal development. If there is a lack of food and/or overcrowding at the end of L1, the larva will enter a developmental program that results in entry into the dauer stage, a morphologically distinct alternative to L3. When food is re-encountered, the dauer larvae will enter L4, resuming normal development. Click here for file

## Figures and Tables

**Table 1:** Translocation of DAF-16 to cytoplasm in ageing worms. Adult worms containing the *muIs71 *DAF-16a::GFP transgene and either wild type or the *hx546 *mutant allele at the *age-1 *locus were kept in isolated cohorts at 20°C and scored for DAF-16::GFP localisation using a fluorescent compound microscope. Nuclei were scored as either distinctly visible or not distinguishable from the cytoplasm. See Figure 6 for images.

*age-1 allele*		No. of worms					
	DAF-16 localisation	DAY 2	DAY 4/5	DAY 6/7	DAY 8/9	DAY 11	DAY 14
wild type	distinct nuclei	28	13	5	1		
	nuclei not visible	0	12	8	16		
							
*age-1(hx546)*	distinct nuclei	18	19	6	29	14	13
	nuclei not visible	0	0	0	0	0	0
