# Supplementary figures and images for: Long-term starvation and ageing induce AGE-1/PI 3-kinase-dependent translocation of DAF-16/FOXO to the cytoplasm
Source: BMC Biol. 2006 Feb 3;4:1. doi: 10.1186/1741-7007-4-1 (PMC1403811; doi:10.1186/1741-7007-4-1)

egg/embryo

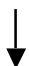

L1

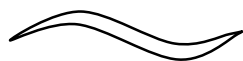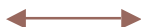

L1 diapause

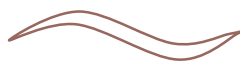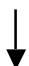

L2

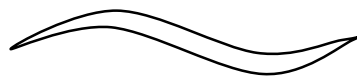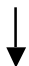

L3

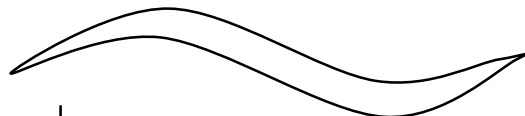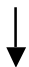

L4

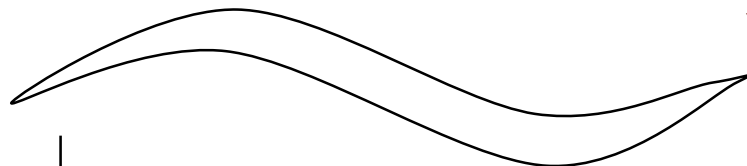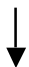

adult

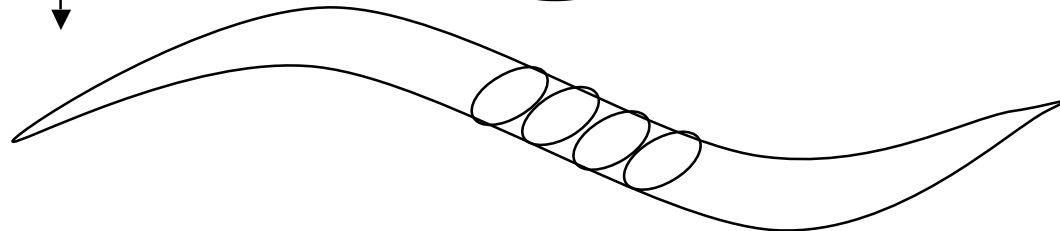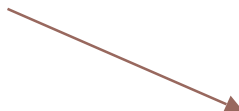

dauer diapause

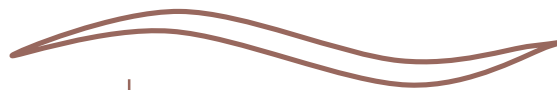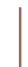

Supplement: Additional File 1 — Diagram depicting the life cycle of C. elegans in the presence of food and in response to starvation. The first stage larva hatches from the eggshell. In the presence of food the larva continues through four larval stages until it becomes a fertile adult. If the larva hatches in the absence of food it will enter the L1 diapause without undergoing the cell division cycles that normally occur in the L1 stage. When food is encountered, the larva will re-enter normal development. If there is a lack of food and/or overcrowding at the end of L1, the larva will enter a developmental program that results in entry into the dauer stage, a morphologically distinct alternative to L3. When food is re-encountered, the dauer larvae will enter L4, resuming normal development. [file 1741-7007-4-1-S1.pdf]
